# Expanding the diversity of Desulfovibrio from the human gut: Desulfovibrio intestinivivus sp. nov., Desulfovibrio desulfopostgatei sp. nov. and Desulfovibrio faecalis sp. nov

**DOI:** 10.1099/ijsem.0.007296

**Published:** 2026-09-24

**Authors:** Marnie Barham, Melinda J. Mayer, Kathryn Gotts, Arjan Narbad, Lizbeth Sayavedra

**Affiliations:** 1 Quadram Institute Bioscience, Norwich Research Park, Norwich NR4 7UQ, UK

**Keywords:** human gut, microbiome, sulphate-reducing bacteria

## Abstract

Three strictly anaerobic, Gram‐negative, curved rod‐shaped bacteria, designated strains QI0430^T^, QI0434^T^ and QI0442^T^, were isolated from faecal samples of healthy adult donors in the UK. Phylogenomic analysis based on 31 single-copy marker genes showed that all three isolates formed distinct lineages. Whole-genome average nucleotide identity and digital DNA–DNA hybridization values between each isolate and its nearest phylogenetic neighbours were <95.6 and <63%, respectively, supporting their status as separate species. Genome sizes ranged from 3.41 to 3.59 Mb, with DNA G+C contents of 56.91–57.43 mol%. QI0430 was a non-motile, rod-shaped, catalase-negative bacterium that grew optimally at 37 °C, pH 7.5 and 0% (wt/v) NaCl. QI0434 was a motile, rod-shaped, catalase-negative bacterium that grew optimally at 30 °C, pH 7.0–8.0 and 0–0.9% (wt/v) NaCl. QI0442 was a non-motile, rod-shaped, catalase-negative bacterium that grew optimally at 30–37 °C, pH 9.0 and optimum 0% (wt/v) NaCl. Based on phylogenomic, phenotypic and chemotaxonomic differences, the following three novel species are proposed: *Desulfovibrio intestinivivus* sp. nov. (type strain QI0430^T^=DSM 120530=NCIMB 15620^T^), *Desulfovibrio desulfopostgatei* sp. nov. (type strain QI0434^T^=DSM 120532=NCIMB 15621^T^) and *Desulfovibrio faecalis* sp. nov. (type strain QI0442^T^=DSM 120531=NCIMB 15622^T^).

## Introduction

Sulphate-reducing bacteria (SRB) are anaerobic micro-organisms that obtain energy through dissimilatory sulphate reduction, producing hydrogen sulphide (H_₂_S) as a byproduct of their metabolism. SRB are taxonomically diverse and inhabit a wide range of environments such as freshwater, soils and the mammalian gut [1]. In the gut, H_2_S plays a dual role: at physiological levels, it acts as a signalling molecule involved in cytoprotection and epithelial energy homeostasis [2]. However, at elevated concentrations, it becomes cytotoxic and genotoxic, disrupting the mucus barrier, impairing epithelial DNA repair pathways and promoting inflammation, which is believed to contribute to the pathogenesis of inflammatory bowel disease and colorectal cancer [3]. H_₂_S-producing SRB have also been shown to interact with prominent gut commensals such as *Bacteroides thetaiotaomicron in vitro* [4] and to modulate host physiology and barrier function *in vivo* [5]. SRB-enriched bacterial consortia from the mucosa of ulcerative colitis (UC) patients have been shown to induce apoptosis in intestinal epithelial cell lines, whereas those from healthy controls do not, supporting a causal link between SRB activity and inflammation [6]. Members of the genus *Desulfovibrio* are the predominant SRB in the human gut, representing over 60% of detected SRB [7, 8], and increases in their abundance have been associated with UC, Crohn’s disease, systemic sclerosis, Parkinson’s disease and irritable bowel syndrome [6, 9–13].

To date, only a handful of *Desulfovibrio* species, such as *D. piger*, *D. fairfieldensis*, *D. legallii*, *D. falkowii, D. intestinalis, D. simplex* and *D. desulfuricans*, have been isolated from the human intestinal tract, despite metagenomic surveys revealing a much greater diversity of *Desulfovibrio* phylotypes [14–19]. Because so few type strains have been properly described, automated taxonomic classifiers frequently misassign *Desulfovibrio* sequences to existing species, masking the true diversity of this genus in the gut microbiome. Consequently, our understanding of the ecological roles and health associations of gut *Desulfovibrio* is limited by the paucity of cultured representatives and high-quality reference genomes.

In this study, three *Desulfovibrio* strains were isolated from faecal samples of healthy adults in the UK. Their taxonomic status was assessed using a combination of phylogenomics, whole-genome average nucleotide identity (ANI), digital DNA–DNA hybridization (dDDH), phenotypic characterization and chemotaxonomic analyses. Together, these data support the proposal of three novel species within the genus *Desulfovibrio*: *Desulfovibrio intestinivivus*, *Desulfovibrio desulfopostgatei* and *Desulfovibrio faecalis*.

## Methods

### Isolation

Strain QI0430^T^ was isolated from a healthy donor aged 25–54. Strains QI0434^T^ and QI0442^T^ were isolated from two healthy donors over the age of 60, with sample collection as described by [20]. All strains were isolated in Norwich, UK (52.621127, 1.218320) in October 2022. Fresh stool samples were processed under anaerobic conditions as soon as they were received. Stool samples were diluted to a final concentration of 0.3 mg ml^−1^ in anaerobic Postgate C medium [14]. The medium contained the following (per litre of ultra-pure water): 6 g sodium lactate, 4.5 g Na_2_SO_4_ (sodium sulphate), 1 g NH_4_Cl, 1 g yeast extract (Oxoid), 0.5 g KH_2_PO_4_, 0.3 g sodium citrate, 0.06 g MgSO_4_.7H_2_O, 1 ml FeSO_4_.7H_2_O (0.4 g in 100 ml H_2_O), 0.04 g CaCl_2_.2H_2_O, 0.5 g cysteine HCl and 4 ml resazurin (0.2 g l^−1^). Cultures were incubated anaerobically at 37** **°C until a black precipitate formed. Once a precipitate was observed, the culture was streaked onto a reduced agar plate of Postgate C. Once colonies formed on the plates, they were sub-cultured three to four more times until similar and uniform colonies were observed. Once the colonies appeared pure, they were picked for 16S rRNA gene sequencing for identification. For long-term storage, strains were stored at −80 °C in Protect Select – Anaerobes tubes (Technical Service Consultants Ltd, UK).

### Whole-genome sequencing and phylogenomic analysis

Sequencing was performed at Quadram Institute Bioscience (Norwich, UK). For short-read sequencing, genomic DNA was extracted using the Maxwell^®^ RSC Blood DNA Kit (Promega, UK) and normalized to 5 ng µl^−1^ with elution buffer (10 mM Tris-HCl). Libraries were prepared in-house as previously described [4] and sequenced on either an Illumina NextSeq 500 or NextSeq 2000 platform. For long-read sequencing, DNA was extracted using the Fire Monkey High Molecular Weight DNA extraction kit (RevoluGen, UK) with an overnight elution. Sequencing was performed with native barcoding genomic DNA using a MinION platform (Oxford Nanopore, UK).

Quality trimming of the Illumina reads was performed using BBDuk (v39.01) [21]. Hybrid assembly was done using Unicycler (v0.5.0) [22]. Contigs were scaffolded with a scaffolding algorithm based on long reads and contig classification (SLR) [23], and assemblies were polished using Pilon (v1.23) [24]. Genome statistics were calculated with Bacterial and Viral Bioinformatics Resource Center (BV-BRC) [25]. Contamination was estimated with CheckM (v1.2.0) [26, 27]. The bacteria were taxonomically classified using GTDB-Tk (v2.1.1) [28, 29].

Reference genomes for members of the *Desulfovibrionaceae* were downloaded from BV-BRC [25] and NCBI and analysed together with genomes generated from strains isolated in this study. Strains designated with the prefix ‘QI’ represent isolates maintained in the Quadram Institute culture collection. A phylogenomic tree was reconstructed using the Bacterial Genome Tree Service from BV-BRC [25]. Single-copy protein families (PGFams) were selected. Aligned proteins and coding DNA from 100 single-copy genes were analysed using RAxML to reconstruct a maximum-likelihood tree. Pairwise ANI and average amino acid identity of *D. desulfuricans* strains were calculated with FastANI (v1.33) and EzAAI (v1.2.2), respectively [30, 31]. dDDH was calculated using the Type (Strain) Genome Server. We only show the results of the formula d_4_, which sums all identities found in high-scoring segment pairs (HSPs) divided by overall HSP length [32]. This formula overcomes issues with incomplete genome assemblies. Genome accession numbers and pairwise ANI and dDDH values for representative strains are provided in Table S1 (available in the online Supplementary Material).

### Cell morphology

Phenotypic characterization was carried out in Postgate C medium [14]. Growth of the cells was assessed at 20, 25, 30, 37 and 42 °C for 5 days. Salt tolerance was tested in medium containing 0, 0.9, 2, 3 and 4% (wt/v) NaCl for 5 days, and pH tolerance was assessed at pH 4, 5, 6, 7, 7.5, 8 and 9 under the same incubation period. Catalase activity was assessed after the addition of 15% (v/v) hydrogen peroxide to cells collected from a freshly grown colony. Gram staining was carried out using a commercial kit (Remel, Thermo Fisher, UK) according to the manufacturer’s instructions.

Motility was assessed via both inoculation in semi-solid media and a wet mount method using *Desulfovibrio legallii* DSM 19129 as a positive control. Hungate tubes with Postgate C medium (0.5% agar) were inoculated by stabbing with overnight cultures using disposable inoculating loops and incubated for 6 days at 37 °C. Motility was determined by observing the spread of bacterial cells from the initial stab site. For the wet mount method, 10 µl of overnight culture was dropped onto a glass slide and a coverslip was placed on top. The cells were then immediately visualized.

### Transmission electron microscopy

Overnight cultures were harvested for transmission electron microscopy (TEM) by centrifugation of 2 ml aliquots at 1,500× ***g*** for 1 min and resuspended in 1 ml Postgate C medium. Samples were then applied to a carbon-coated 300-mesh copper grid (Agar Scientific, UK) for 1 min, and excess liquid was removed with filter paper. Grids were negatively stained with 2% (wt/v) uranyl acetate for 1 min, blotted to remove excess stain and air-dried prior to imaging. Samples were imaged using an FEI Talos F200C TEM (Thermo Fisher Scientific, The Netherlands), fitted with a OneView camera (Gatan, USA), operating at 200 kV.

### Carbon source utilization

Carbon utilization analysis was carried out using a Biolog AN microplate (TECHNOPATH Distribution, Ireland) following the manufacturer’s guidelines (00P 003 Rev E – AN Microplate IFU). Briefly, cultures were heavily streaked onto Postgate C agar plates and incubated in an anaerobic cabinet at 37 °C for 1–2 days. Cells were then collected from the plate with a sterile cotton swab and resuspended in the Biolog anaerobe inoculating fluid (AN-IF) to give a turbidity of 0.19–0.20 (OD600). The cell suspension was then taken out of the anaerobic cabinet and used to inoculate the AN microplate. The plates were allowed to stand for 15 min at room temperature before being sealed in an anaerobic bag with a gas-generating sachet. Plates were incubated at 37 °C for 24 h, and reactions were scored visually.

### Fatty acid composition

Cells were cultured in Postgate C medium at 37 °C for 1–2 days, harvested by centrifugation at 1,699 ***g*** for 10 min and freeze-dried. Cellular fatty acid methyl ester analysis was carried out by DSMZ services, Leibniz-Institut DSMZ – German Collection of Microorganisms and Cell Cultures GmbH (Braunschweig, Germany).

## Results

### Genomic and phylogenomic context

**Fig. 1.**
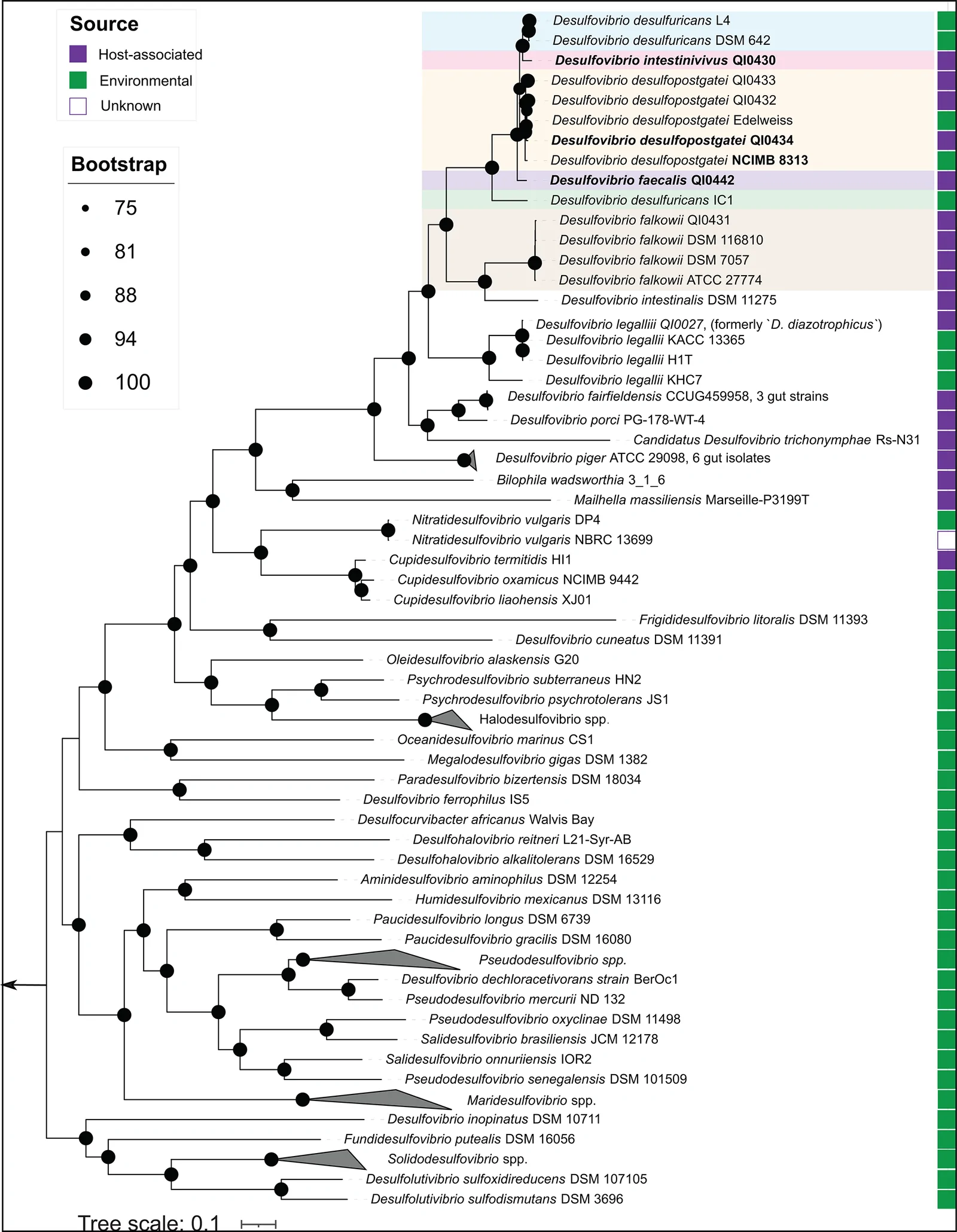
Maximum-likelihood phylogenomic tree reconstructed from a concatenation of 100 single-copy marker genes using RAxML. The tree was rooted with *Ruminiclostridium cellobioparum* subsp. *termitidis* CT1112. Newly isolated strains proposed as novel species in this study are highlighted in bold. Highlighted clades indicate where strains were previously classified as *D. desulfuricans* or newly isolated strains. Strains with the prefix ‘QI’ were isolated as part of this study.

**Fig. 2.**
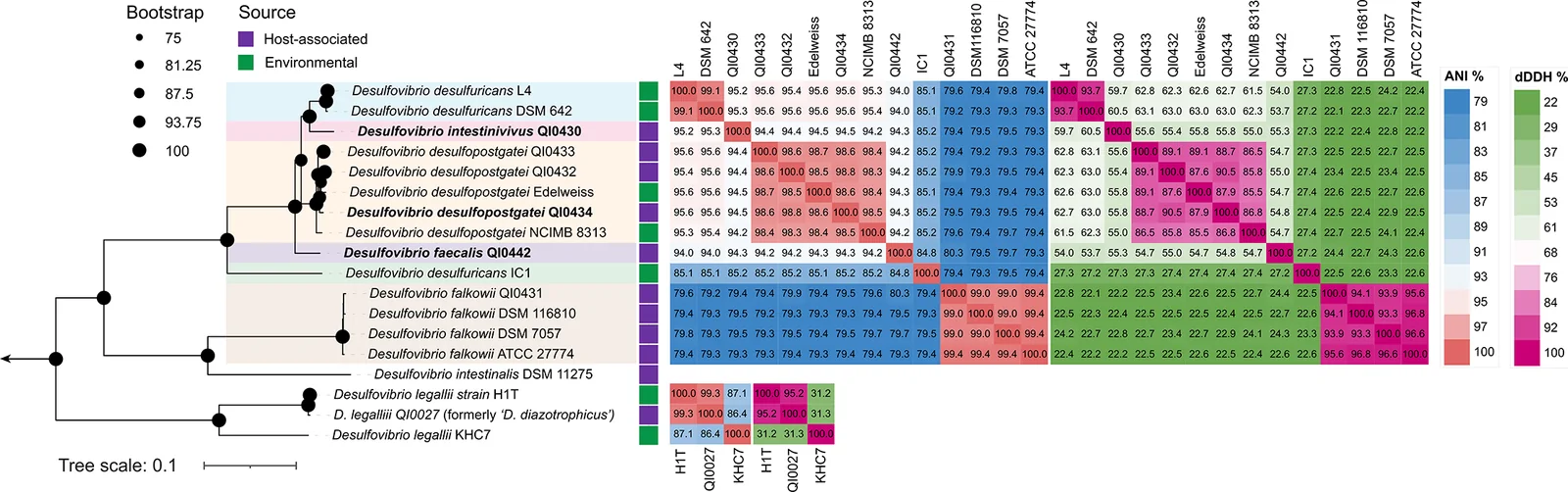
Maximum-likelihood phylogenomic tree reconstructed from a concatenation of 100 single-copy marker genes using RAxML. The tree was rooted with *R. cellobioparum* subsp. *termitidis* CT1112. Heatmaps represent ANI and dDDH estimated with formula d_4_. Highlighted clades indicate where strains were previously classified as *D. desulfuricans* or newly isolated strains. Strains with the prefix ‘QI’ were isolated as part of this study.

Assembled genome sequences of the QI0430^T^, QI0434^T^ and QI442^T^ were 3.46, 3.41 and 3.59 Mb, respectively (Table 1). Genome completeness was 100% for all strains, while genome contamination ranged from 0.00 to 0.40%, a range commonly observed when sequencing pure cultures. Phylogenomic analysis revealed six distinct clades amongst strains designated as *Desulfovibrio desulfuricans* together with the newly isolated strains (Fig. 1), indicating that reclassification within this group is warranted. Following currently accepted species thresholds – ANI<95–96% or dDDH<70% [33, 34] – all three isolates met the criteria for novel species (Fig. 2). Strain QI0430^T^ formed its own clade, showing 95.3% ANI and 60.5% dDDH to the *D. desulfuricans* DSM 642 type strain. Strain QI0434^T^, assigned to clade 3, had ANI and dDDH values of 95.6 and 63%, respectively, with the *D. desulfuricans* DSM 642 type strain and grouped with isolates from both host-associated and environmental sources. Strain QI0442^T^ also formed its own clade and had ANI and dDDH values of 94.0 and 53.7%, respectively. Together, our results support the classification of QI0430^T^, QI0434^T^ and QI0442^T^ as representing three novel species within the genus *Desulfovibrio*.

**Table 1.** Genome features of the three isolated strains QI0430^T^, QI0434^T^ and QI0442^T^, and the type strain of the closely related *D. desulfuricans* DSM 642

Strain	DSM 642**^T^**	QI0430^T^	QI0434^T^	QI0442^T^
**Genome statistics**				
Genome size (bp)	3,391,683	3,460,865	3,409,866	3,593,866
G+C (mol%)	57.4	57.09	57.43	56.91
Number of contigs	20	2	2	5
N_50_	378,826	3,384,455	3,408,615	3,562,259
Genome completeness (%)	100.00	100.00	100.00	100.00
Genome contamination (%)	0.2	0.40	0.00	0.20
**Annotation statistics**				
Number of tRNAs	51	55	55	64
Number of rRNAs	4	9	9	9
Number of coding sequences	3123	3287	3253	3516
Number of clustered regularly interspaced short palindromic repeat (CRISPR) arrays	1	2	1	1
Presence of dissimilatory sulphate-reduction genes (*sat, aprAB, dsrABD, dsrC, qmoABC* and *dsrMKJOP)*	Yes	Yes	Yes	Yes

Hamaguchi *et al.* [18] recently described *Desulfovibrio falkowii*. Our genomic analyses indicate that strains DSM 7057 and ATCC 27774 fall within the genomic species boundary of *D. falkowii*, sharing >99% ANI with the type strain. These strains are therefore best interpreted as additional representatives of this species. Additionally, *D. desulfuricans* IC1 also falls below the species delineation thresholds and would be classified as a separate species; however, as it is preserved in only a single culture collection, we did not formally reclassify it here.

Additionally, QI0027 was previously described as the novel species *Desulfovibrio diazotrophicus* [14]. However, the original comparison was based on the publicly available genome KHC7, which at the time was attributed to *D. legallii*. Subsequent availability of genome sequences from the authenticated *D. legallii* type-strain lineage, H1ᵀ demonstrated that strain QI0027 falls within the genomic species boundary of *D. legallii*. ANI analysis between QI0027 and H1ᵀ was 99.3%, and dDDH was 95.2%. These values are above the accepted thresholds for species delineation and indicate that QI0027 belongs to *D. legallii*. Therefore, strain QI0027 should be regarded as a member of *D. legallii*, despite the phenotypic differences previously described [14], including the absence of motility and flagella in QI0027 under the conditions examined.

In contrast, strain KHC7 was clearly separated from the *D. legallii* type-strain lineage in both ANI and dDDH analyses (Fig. 2). ANI values between KHC7, QI0027 and H1ᵀ were ~86–87%, and the dDDH value between KHC7 and QI0027 was 31.3%, all substantially below accepted species delineation thresholds. These results indicate that KHC7 does not belong to *D. legallii*. However, as KHC7 is not currently available from recognized culture collections and no comparative phenotypic characterization was undertaken, no formal taxonomic reassignment is proposed here.

### Phenotypic characterization

All three strains were Gram-negative and catalase-negative. Black precipitate formation consistent with iron sulphide formation was observed across all three strains. QI0430^T^ grew at temperatures of 20–37 °C, tolerated 0–2% (wt/v) NaCl concentrations and pH 5–9. QI0442^T^ grew at temperatures of 20–37 °C, tolerated 0–2% (wt/v) NaCl concentrations and pH 6–9. QI0434^T^ grew at 20–42 °C, tolerated 0–3% NaCl and pH 5–9 (Table 2). All strains had light brown, circular colonies with entire margins and a slightly raised elevation on Postgate C agar after 4 day incubation at 37 °C.

**Table 2.** Phenotypic characterization of QI0430, QI0434, QI0442 and DSM 642. Optimal growth conditions are in brackets next to the growth range

Characteristic	DSM 642	QI0430	QI0434	QI0442
Catalase activity	nd	Negative	Negative	Negative
Gram stain	Negative	Negative	Negative	Negative
Colony colour on Postgate C agar	Light brown	Light brown	Light brown	Light brown
Black precipitate formation	Yes	Yes	Yes	Yes
Motility	Yes [18]	No	Yes	No
Cell length (µm)	2.1–2.8	1.2–2.9	1.4–3.2	2.0–4.8
Temperature range for growth (°C)	nd	20–37 (37)	20–42 (30)	20–37 (30–37)
NaCl range for growth (%)	0–2.4 [18]	0–2 (0)	0–3 (0–0.9)	0–2 (0)
pH range for growth	nd*	5–9 (7.5)	5–9 (7–8)	6–9 (9)
Source	Tar and sand mix around corroded gas main	Human faeces	Human faeces	Human faeces

*Unknown data are represented with not determined (ND).

**Fig. 3.**
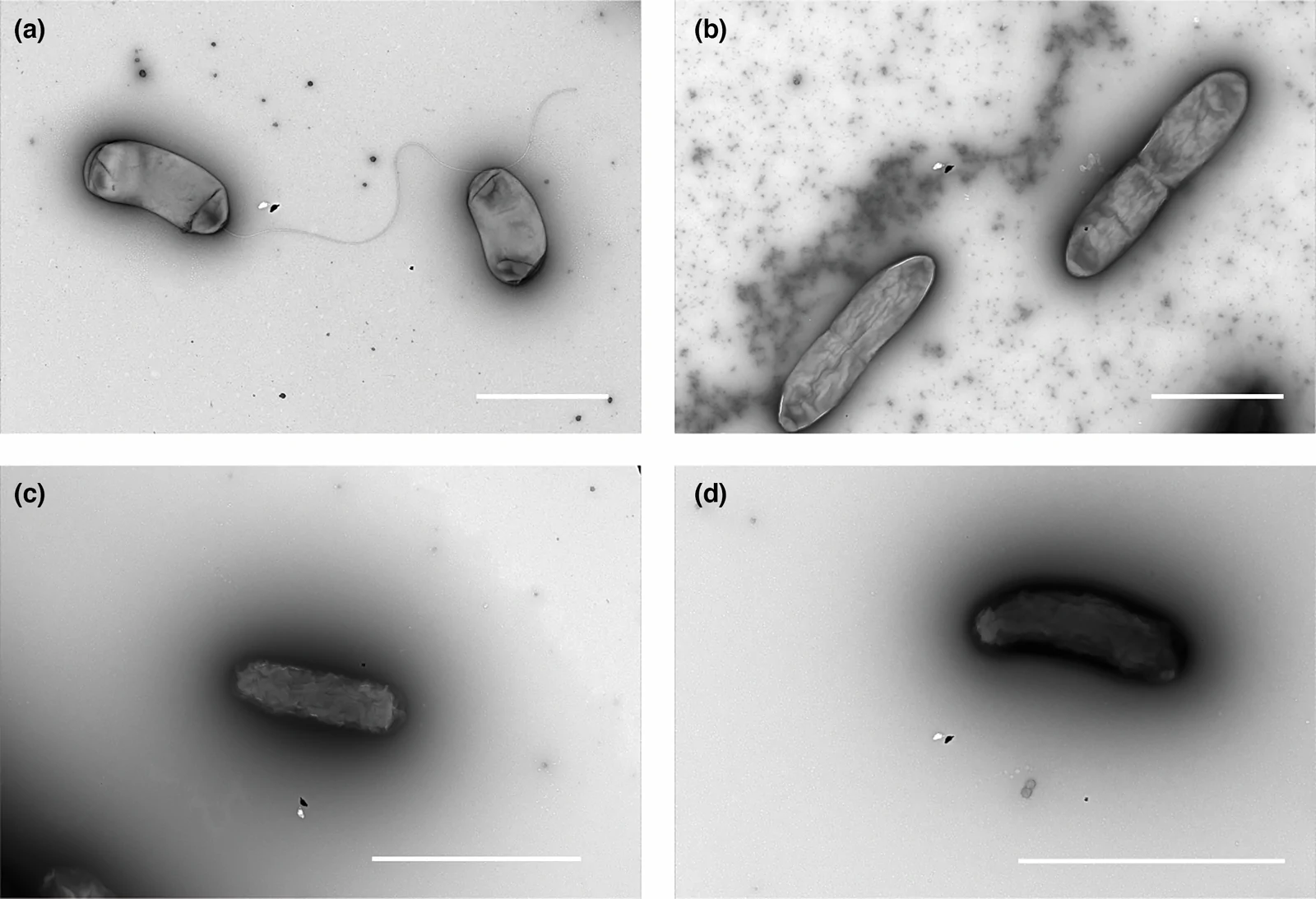
Transmission electron micrographs of *Desulfovibrio* strains: reference strain DSM 642 (**a**), and novel isolates QI0442T (**b**), QI0434T (**c**) and QI0430T (**d**). Scale bars=2 µm.

Based on both soft agar and wet mount motility assays QI0430^T^ and QI0442^T^ were non-motile, while QI0434 was motile; however, no flagella were observed for any strain by TEM using negative staining (Figs 3 and S1). Motility is generally considered a characteristic feature of the genus *Desulfovibrio*, with *D. piger* being one of the few reported exceptions. Interestingly, strains QI0430ᵀ and QI0442ᵀ were consistently non-motile in both semi-solid agar and wet mount assays. Although no flagella were observed by TEM, the possibility that flagella were lost during sample preparation cannot be excluded. A similar discrepancy was observed previously within the *D. legallii* lineage, where strain QI0027 lacked motility, and no flagella were observed by TEM, whereas the type strain *D. legallii* DSM 19129 is motile [14]. These observations suggest that motility may be more variable among host-associated *Desulfovibrio* than currently appreciated. One possible explanation is that expression of flagellar genes is regulated in response to environmental conditions or nutrient availability [35]. Alternatively, the flagellar system may no longer be functional despite retention of the underlying genes. Such variability may reflect adaptation to a relatively stable, nutrient-rich environment where active motility confers less selective advantage. However, additional isolates, together with comparative genomic and transcriptomic analyses, will be required to determine whether the loss of motility is a common feature among host-associated members of the genus and whether it results from regulatory or structural changes.

All three strains utilized d-galacturonic acid, d-galactose, l-rhamnose, palatinose, l-fucose, d-lactic acid methyl ester, d-mannose, α-d-glucose, turanose, 3-methyl-d-glucose, pyruvic acid, d,l-lactic acid, α-hydroxybutyric acid and α-ketobutyric acid (Table 3). DSM 642 was also able to use all of these substrates except α-d-glucose. In addition, QI0430^T^ can also utilize d-fructose, pyruvic acid methyl ester and d-cellobiose; QI0434^T^ can utilize d-fructose, pyruvic acid methyl ester, l-serine and l-malic acid; and QI0442^T^ can utilize d-raffinose and succinic acid. DSM 642 also used d-fructose, pyruvic acid methyl ester, l-serine and l-malic acid, in addition to a number of other substrates that were not utilized by the three gut strains.

**Table 3.** Carbon source utilization profiles of DSM 642, QI0434, QI0430 and QI0442 determined using Biolog AN microplates. Positive utilization is indicated by ‘+’, while negative utilization is indicated by ‘−’. For variable results amongst replicates, the number of positive reactions was presented relative to the total replicates

Substrate	DSM 642	QI0434	QI0430	QI0442
d-Galacturonic acid	+	+	+	+
d-Galactose	+	+	+	+
l-Rhamnose	+	+	+	+
Palatinose	+	+	+	+
l-Fucose	+	+	+	+
d-Lactic acid methyl ester	+	+	+	+
α-d-Glucose	−	+	+	+
d-Mannose	+	+	+	+
3-Methyl-d-glucose	+	+	+	+
Pyruvic acid	+	+	+	+
d,l-Lactic acid	+	+	+	+
α-Hydroxybutyric acid	+	+	+	+
α-Ketobutyric acid	+	+	+	+
Turanose	+	+	+	+
d-Fructose	+	+	+	−
Pyruvic acid methyl ester	+	+	+	−
d-Cellobiose	−	−	+	−
d-Raffinose	−	−	−	+
Succinic acid	−	−	−	+
l-Serine	+	+	−	−
l-Malic acid	+	+	−	−
2′-Deoxyadenosine	+	−	−	−
d,l-α-Glycerol phosphate	+	−	−	−
Gentiobiose	+	−	−	−
Fumaric acid	+	−	−	−
l-Phenylalanine	+	−	−	−
Acetic acid	+	−	−	−
d-Glucosaminic acid	+	−	−	−
d-Gluconic acid	+	−	−	−
Inosine	+	−	−	−
Arbutin	2/3	−	−	−
d-Melibiose	2/3	−	−	−
d-Glucose-6-phosphate	2/3	−	−	−
myo-Inositol	2/3	−	−	−
meso-Tartaric acid	2/3	−	−	−
l-Lactic acid	2/3	−	−	−
α-d-Glucose-1-phosphate	2/3	−	−	−

### Chemotaxonomic characterization

The major cellular fatty acids comprising over 10% in QI0430^T^: C_16 : 0_ (26.8%), iso-C_17 : 1_ ω9c (24.1%), C_16 : 1_ ω9c (13.8%) and iso-C_15 : 0_ (13.1%); in QI0434^T^, C_16 : 0_ (27.5%), iso-C_17 : 1_ ω9c (22.8%), C_16 : 1_ ω9c (15.4%) and iso-C_15 : 0_ (11.0%); and in QI0442^T^, C_16 : 0_ (37.3%), iso-C_17 : 1_ ω9c (14.0%), iso-C_15 : 0_ (13.5%) and C_16 : 1_ ω9c (10.8%).

## Description of *Desulfovibrio intestinivivus* sp. nov.

*Desulfovibrio intestinivivus* (in.tes.ti.ni.vi'vus. L. neut. *n. intestinum*, intestine; L. masc. *adj. vivus*, alive, living; N.L. masc. *adj. intestinivivus*, living in the intestine). Cells are 1.2–2.9 µm long rods and are anaerobic, non-motile, catalase-negative and Gram-negative. Growth occurs at 20–37 °C (optimum 37 °C), pH 5.0–9.0 (optimum pH 7.5) and in the presence of 0–2% (wt/v) NaCl (optimum 0%). Colonies on Postgate C agar after 4 days at 37 °C are light brown, circular, slightly raised and have entire margins. The major cellular fatty acids are C_16 : 0_, iso-C_17 : 1_ ω9c, C_16 : 1_ ω9c and iso-C_15 : 0_. QI0430^T^ can utilize d-galacturonic acid, d-galactose, l-rhamnose, palatinose, l-fucose, d-lactic acid methyl ester, d-mannose, α-d-glucose, turanose, 3-methyl-d-glucose, pyruvic acid, d,l-lactic acid, α-hydroxybutyric acid, α-ketobutyric acid, d-fructose, pyruvic acid methyl ester and d-cellobiose.

The type strain QI0430^T^ (=DSM 120530,=NCIMB 15620) was isolated from human faeces. The GenBank accession numbers are PX377545 for the 16S rRNA gene sequence and JBRBQT000000000 for the genome sequence. The genomic G+C content is 57.09 mol%.

## Description of *Desulfovibrio desulfopostgatei* sp. nov.

*Desulfovibrio desulfopostgatei* (de.sul.fo.post.ga'te.i. N.L. pref. *desulfo*-, referring to a sulphate-reducing prokaryote; N.L. gen. n. *postgatei*, of Postgate, named in honour of Professor John R. Postgate (1922–2014), pioneer in the biology of sulphate-reducing bacteria; N.L. gen. n. *desulfopostgatei*, a sulphate-reducing bacterium named in honour of Professor John R. Postgate). Cells are 1.4–3.2 µm long rods and are anaerobic, motile, catalase-negative and Gram-negative. Growth occurs at 20–42 °C (optimum 30 °C), pH 5.0–9.0 (optimum pH 7.0–8.0) and in the presence of 0–3% (wt/v) NaCl (optimum 0–0.9%). After 4 days on Postgate C agar at 37 °C, colonies are light brown, circular, slightly raised and possess entire margins. The major cellular fatty acids are C_16 : 0_, iso-C_17 : 1_ ω9c, C_16 : 1_ ω9c and iso-C_15 : 0_. QI0434^T^ can utilize d-galacturonic acid, d-galactose, l-rhamnose, palatinose, l-fucose, d-lactic acid methyl ester, d-mannose, α-d-glucose, turanose, 3-methyl-d-glucose, pyruvic acid, d,l-lactic acid, α-hydroxybutyric acid, α-ketobutyric acid, d-fructose, pyruvic acid methyl ester, l-serine and l-malic acid.

The type strain QI0434^T^ (=DSM 120532,=NCIMB 15621) was isolated from human faeces. The GenBank accession numbers are PX377543 for the 16S rRNA gene sequence and JBRBQS000000000 for the genome sequence. Genomic G+C content is 57.43 mol%.

## Description of *Desulfovibrio faecalis* sp. nov.

*Desulfovibrio faecalis* (fae.ca'lis. L. masc. *adj. faecalis*, pertaining to faeces, referring to the source of isolation). Cells are 2.0–4.8 µm long rods and are anaerobic, non-motile, catalase-negative and Gram-negative. Growth occurs at 20–37 °C (optimum 30–37 °C), pH 6.0–9.0 (optimum pH 9.0) and in the presence of 0–2% (wt/v) NaCl (optimum 0%). After 4 days on Postgate C agar at 37 °C, colonies are light brown, circular, slightly raised and have entire margins. The major cellular fatty acids are C_16 : 0_, iso-C_17 : 1_ ω9c, iso-C_15 : 0_ and C_16 : 1_ ω9c. QI0442ᵀ can utilize d-galacturonic acid, d-galactose, l-rhamnose, palatinose, l-fucose, d-lactic acid methyl ester, d-mannose, α-d-glucose, turanose, 3-methyl-d-glucose, pyruvic acid, d,l-lactic acid, α-hydroxybutyric acid, α-ketobutyric acid, d-raffinose and succinic acid.

The type strain QI0442ᵀ (=DSM 120531ᵀ,=NCIMB 15622ᵀ) was isolated from human faeces. The GenBank accession numbers are PX377544 for the 16S rRNA gene and JBRBQR000000000 for the whole-genome sequence. The genomic DNA G+C content is 56.91 mol%.

## Supplementary material

10.1099/ijsem.0.007296 Supplementary Material 1.

10.1099/ijsem.0.007296 Supplementary Material 2.

## Abbreviations

ANI: average nucleotide identity
BV-BRC: Bacterial and Viral Bioinformatics Resource Center
CRISPR: clustered regularly interspaced short palindromic repeats
dDDH: digital DNA–DNA hybridization
H_₂_S: hydrogen sulphide
HSP: high-scoring segment pair
SRB: Sulphate-reducing bacteria
TEM: transmission electron microscopy
UC: ulcerative colitis
