# Supplementary material for: Expanding the diversity of Desulfovibrio from the human gut: Desulfovibrio intestinivivus sp. nov., Desulfovibrio desulfopostgatei sp. nov. and Desulfovibrio faecalis sp. nov
Source: Int J Syst Evol Microbiol. 2026 Sep 24;76(9):007296. doi: 10.1099/ijsem.0.007296 (PMC13609909; doi:10.1099/ijsem.0.007296)
Supplement: Supplementary file 1 [file ijsem-76-07296-s002.pdf]

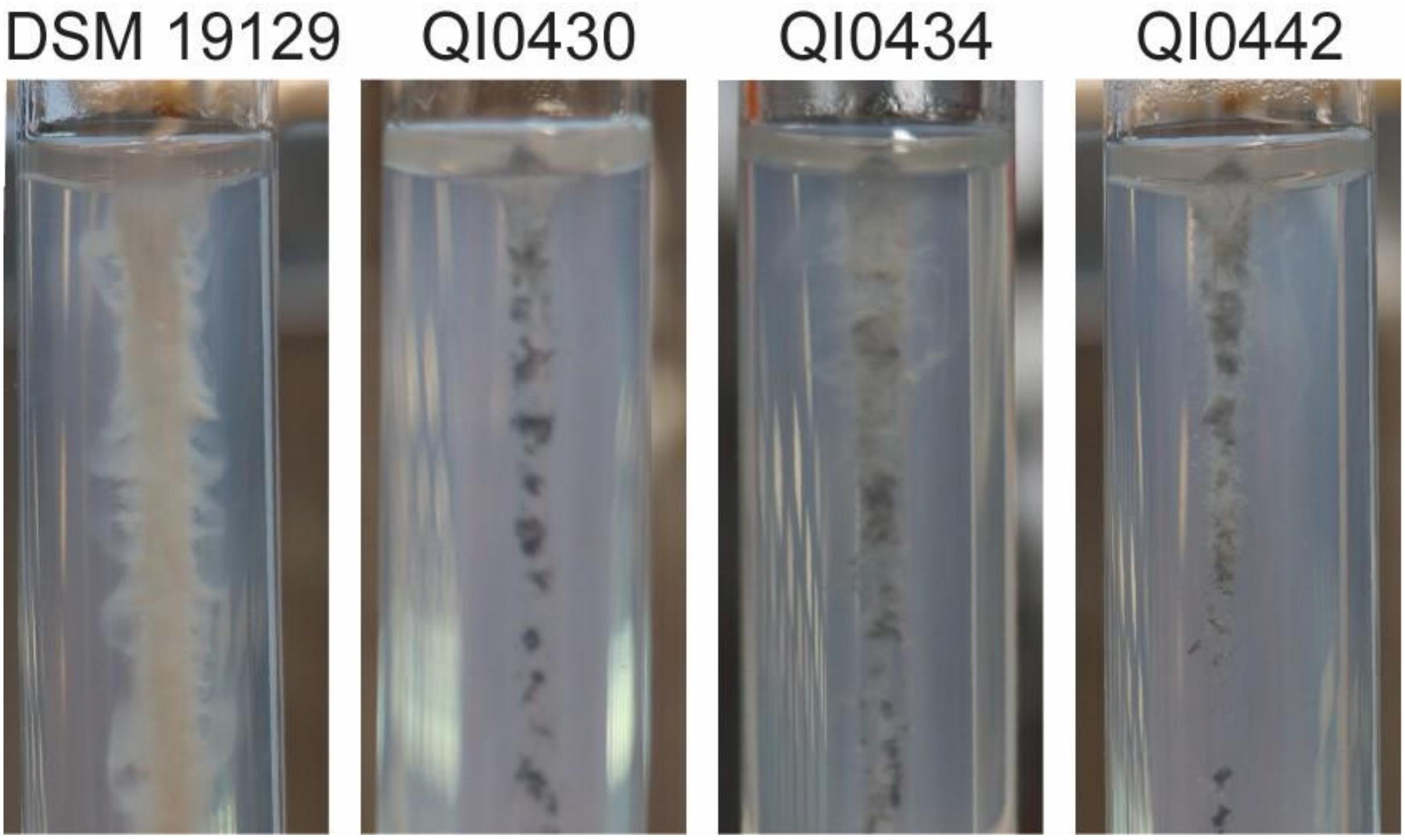

Supplementary Figure 1. Motility assays with *D. legallii* DSM 19129 as a positive control. Bacteria motility is shown by cloudy growth coming away from the stab site, as seen in DSM 19129 and QI0434. Non-motile bacteria show a clear edge at the stab site as seen with QI0430 and QI0442.
